# Engineering TCR-directed T-cells for the treatment of multiple myeloma

**DOI:** 10.3389/fimmu.2026.1755096

**Published:** 2026-03-31

**Authors:** Nida Mubin, James J. Ignatz-Hoover, James J. Driscoll

**Affiliations:** 1 Division of Hematology & Oncology, Case Western Reserve University, Cleveland, OH, United States; 2 Adult Hematologic Malignancies & Stem Cell Transplant Section, Seidman Cancer Center, University Hospitals Cleveland Medical Center, Cleveland, OH, United States; 3 Case Comprehensive Cancer Center, Case Western Reserve University, Cleveland, OH, United States

**Keywords:** CAR T-cell, drug resistance, multiple myeloma, proteasome-dependent MHC class I antigen, TCR-engineered T-cell immunotherapy

## Abstract

Multiple myeloma (MM) is a virtually incurable plasma cell malignancy characterized by malignant cells that expand within the tumor-permissive bone marrow (BM) microenvironment. Novel strategies are urgently needed to improve the outcomes of patients with difficult-to-treat and therapy-refractory disease. The ability to genetically manipulate T-cells and the introduction of adoptive cellular therapies (ACTs) has improved the treatment of relapsed and/or refractory (RR)MM. Emerging evidence supports the efficacy of ACTs as early lines of cancer treatment, potentially even as an alternative to autologous hematopoietic stem cell transplantation. Chimeric antigen receptor (CAR) T-cell therapies based upon genetically engineered patient-derived T-cells are utilized in routine clinical practice, however severe toxicities, therapeutic resistance, exorbitant costs, a cumbersome manufacturing process and production logistics limits their broader application. Tumor-infiltrating lymphocytes (TILs) can also mediate tumor regression and lead to durable responses, but wider efficacy is restricted by limited accessibility, reduced proliferative capacity and low effector function. In this context, autologous T-cells engineered to express T-cell receptors (TCRs) represent an intriguing option to improve MM treatment. Immunoproteasomes represent an essential cornerstone of adaptive immunity and are required for the efficient processing of antigenic peptides presented by MHC class I (MHC-I) molecules to cytotoxic CD8^+^ T-lymphocytes (CTLs). Recent studies have demonstrated that immunoproteasome activation increases the presentation of tumor-specific neo-antigens, thereby offering a potential strategy to improve the antimyeloma effects of T-cell-mediated immunotherapies. Here, we discuss advantages and strategies that support the administration of TCR-engineered T-cells for the treatment of MM. This review focuses on the role of immunoproteasome dependent antigen processing in shaping the myeloma immunopeptidome and enabling TCR-based immunotherapy. We discuss how modulation of neoantigen presentation may inform the design of TCR-engineered T cells and related immunotherapeutic strategies for MM.

## 1 Introduction

Multiple myeloma (MM) is a plasma cell neoplasm characterized by an atypical increase of monoclonal immunoglobulins that results in end-organ damage, often affecting bones, kidneys, and causing anemia (1–3). MM is the second most common hematologic malignancy and continues to show an increasing global incidence (1–5). MM cells are genetically complex and display a high degree of heterogeneity that develops through the multistep acquisition of genetic alterations within the tumor cells and changes that occur in the bone marrow (BM) microenvironment (6–11). The incidence of MM is ~36,000 new cases annually in the U.S., affecting men more than women, predominantly older adults, with a lifetime risk of <1% (12–17). Non-Hispanic Black individuals have significantly higher rates, and global rates vary, with increasing trends expected. While the advent of novel agents targeting disease biology and autologous stem cell transplantation (ASCT) has improved five-year overall survival (OS) rates, MM continues to carry an increased risk of morbidity and mortality with reduced quality of life (QOL) (18, 19).

Monoclonal gammopathy of undetermined significance (MGUS) is a non-cancerous, proliferative plasma cell disorder and is one of the most common premalignant disorders in Western countries. MGUS has a prevalence of 3.2% in Caucasians over 50 years of age and is associated with a life-long risk of progression to MM (20, 21). In transplant-ineligible or transplant-deferred patients, frontline therapy commonly includes daratumumab, lenalidomide, and dexamethasone, whereas treatment at relapse increasingly incorporates newly approved immunotherapies such as bispecific monoclonal antibodies and chimeric antigen receptor (CAR) T-cell therapy (22–25).

International Myeloma Working Group (IMWG) criteria define symptomatic MM by BM infiltration of ≥10% clonal plasma cells and the presence of at least one myeloma-defining event, including CRAB features or biomarkers of imminent organ damage (26).

Despite approval of more than 20 therapeutic agents by the U.S. Food and Drug Administration (FDA), MM remains difficult to cure, and most patients eventually relapse, with successive remissions decreasing in duration as drug resistance emerges through clonal evolution of surviving myeloma cells (27–30). Cellular and acellular components within the BM microenvironment further promote myeloma survival, while activation of signaling pathways such as AKT, NF-κB, and IL-6 leads to therapeutic failure. In parallel immune dysfunction in MM patients compromises the generation of effective anti-tumor immune response.

T-cell receptors (TCRs) mediate anti-tumor responses by recognizing peptides that are derived from specific tumor antigens or neoantigens (NeoAgs) and presented by Major Histocompatibility Complex (MHC) class I molecules on the surface of cancer cells, leading to cytotoxic CD8^+^ T-cell activation and tumor cell killing. While TCR-mediated recognition of tumor antigens has been well characterized in solid tumors, its role in targeting hematologic malignancies remains less well defined, and the clinical relevance of TCR-directed T cells with anti-myeloma activity is still emerging. Here, we provide an overview of the advantages of TCR-engineered T cells for the treatment of MM, particularly in patients with disease refractory to currently available therapies.

In this review, we discuss the biological rationale for TCR-engineered T-cell based therapeutic potential, and emerging strategies supporting the use of TCR-engineered T cells in MM, with a particular focus on proteasome-dependent antigen processing and its implications for TCR-directed immunotherapy, to enhance the efficacy and safety of TCR-based immunotherapies in patients with refractory disease.

## 2 Adoptive T-cell therapies in multiple myeloma

**Figure 1:**
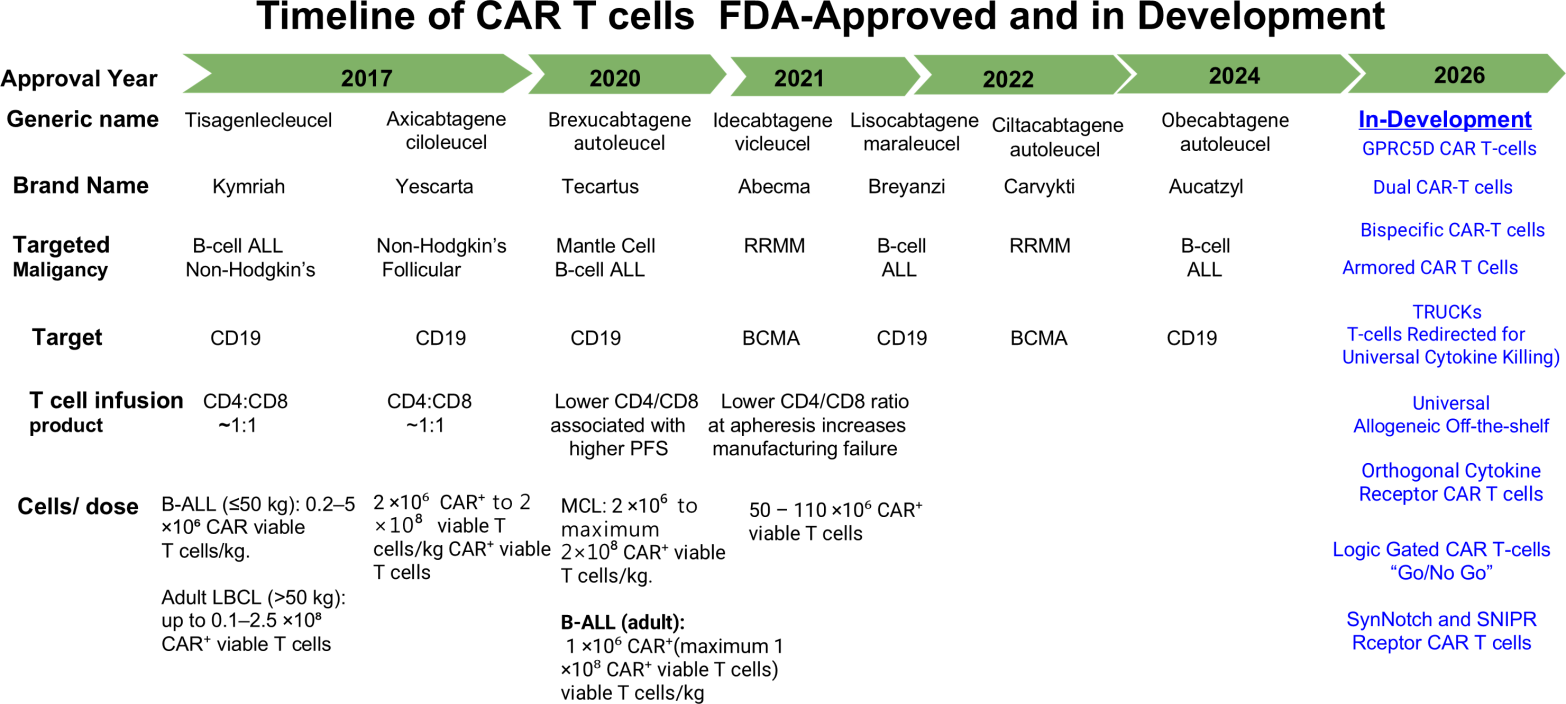
Timeline of CAR T-cells that have been FDA-Approved or are in clinical development for B-cell malignancies; The timeline depicts CAR T-cell products targeting CD19 or B-cell maturation antigen (BCMA), annotated by year of initial FDA approval or clinical development status. ALL, acute lymphoblastic leukemia; BCMA, B-cell maturation antigen; CAR T, chimeric antigen receptor T cell; CLL/SLL, chronic lymphocytic leukemia/small lymphocytic lymphoma; FDA, U.S. Food and Drug Administration; FL, follicular lymphoma; LBCL, large B-cell lymphoma; MCL, mantle cell lymphoma; MM, multiple myeloma.

Immunotherapies generate myeloma-directed cytotoxicity, ideally promoting longitudinal immune memory and improving patient outcomes (31–34). Adoptive cellular therapies (ACT), include using tumor-infiltrating lymphocytes (TILs) or autologous T-cells that are genetically engineered to express CARs or TCRs target tumor-associated antigen. CAR T-cell therapy has emerged as the most clinically established ACT-based modality and has demonstrated significant efficacy in hematologic malignancies, achieving durable remissions in patients with refractory non-Hodgkin lymphoma (NHL), B-cell acute lymphoblastic leukemia (ALL), and MM (**Table 1**). Several CAR T-cell therapies have recently been approved by the U.S. Food and Drug Administration (FDA) (**Figure 1**). Over the past decade, CAR T-cell platforms have undergone multiple iterative refinements, resulting in substantial advances over earlier generations (35, 36).

**Table 1:** FDA-approved CAR T-cells for B cell malignancies.

Brand name	Generic name	Abbreviation	Target	FDA-approval	FDA-approved indication in B-cell malignancies
Obe-cel (Aucatzyl)	Obecabtagene autoleucel	obe-cel	CD19	2024	Adult relapsed/refractory B-cell precursor ALL
Carvykti	Ciltacabtagene autoleucel	cilta-cel	BCMA	2022	Relapsed/refractory MM (≥4 prior lines; expanded to ≥1 prior line in lenalidomide-refractory disease)
Breyanzi	Lisocabtagene maraleucel	liso-cel	CD19	2021 (expanded 2024)	Relapsed/refractory LBCL; FL (≥3L); MCL; CLL/SLL after BTKi and BCL-2i
Abecma	Idecabtagene vicleucel	ide-cel	BCMA	2021 (expanded 2024)	Relapsed/refractory MM (≥4 prior lines; expanded to ≥2 prior lines)
Tecartus	Brexucabtagene autoleucel	brexu-cel	CD19	2020	Relapsed/refractory MCL; adult relapsed/refractory B-cell precursor ALL
Kymriah	Tisagenlecleucel	tisa-cel	CD19	2017	Pediatric and young adult relapsed/refractory B-cell ALL; adult DLBCL
Yescarta	Axicabtagene ciloleucel	axi-cel	CD19	2017	Adult relapsed/refractory LBCL (DLBCL, PMBCL, high-grade B-cell lymphoma, DLBCL arising from FL)

ALL, acute lymphoblastic leukemia; BCMA, B-cell maturation antigen; BCL-2i, B-cell lymphoma 2 inhibitor; BTKi, Bruton tyrosine kinase inhibitor; CLL/SLL, chronic lymphocytic leukemia/small lymphocytic lymphoma; DLBCL, diffuse large B-cell lymphoma; FL, follicular lymphoma; LBCL, large B-cell lymphoma; MCL, mantle cell lymphoma; MM, multiple myeloma; PMBCL, primary mediastinal B-cell lymphoma; RR, relapsed/refractory.

In MM, B-cell maturation antigen (BCMA) has emerged as a key therapeutic target. BCMA is a transmembrane glycoprotein that is highly expressed on malignant plasma cells, promoting plasma cell survival and expansion through binding to its ligands, BAFF (B-cell activating factor) and APRIL (a proliferation-inducing ligand). Two BCMA directed CAR T-cell therapies, idecabtagene vicleucel (ide-cel, Abecma) and ciltacabtagene autoleucel (cilta-cel, Carvykti) are FDA-approved for the treatment of RRMM. Ide-cel consists of autologous T-cells, that have been Lenti virally transduced to express a CAR that binds BCMA through an scFv, whereas cilta-cel incorporates two camelids derived VHH domains for BCMA recognition, coupled with signaling through 4-1BB and CD3ζ cytoplasmic domains. BCMA expression and soluble BCMA levels have been correlated with diseases status and therapeutic response in MM, and additional BCMA-targeting modalities, including bispecific T-cell engagers and antibody–drug conjugates, are under active clinical investigation (37–39). Soluble BCMA (sBCMA) levels have also been shown to decrease during treatment in response to CAR T-cells and may correlate with disease status. Other modalities used to target BCMA include bispecific antibody T-cell engagers (BiTEs) and antibody–drug conjugates (ADCs). Recent clinical studies suggest that BCMA-directed CAR T-cells hold promise to achieve deeper and durable responses and support their investigation as earlier lines of treatment for RRMM (39–44).

Although BCMA-targeted CAR T-cell therapies induce deep initial responses in many patients, disease relapses remain common, underscoring the urgent need for effective post-BCMA treatment strategies. Other surface antigens currently under investigation include SLAMF7 and CD38, however, their expression on non-B-cell lineage tissues raise safety concerns related to on-target, off-tumor toxicity. G protein–coupled receptor class C group 5 member D (GPRC5D) has emerged as a compelling alternative antigen due to its relatively restricted expression pattern. GPRC5D-directed CAR T-cell therapies have recently advanced into clinical testing (**Table 2**) and have demonstrated meaningful clinical activity. Nevertheless, as observed with BCMA-directed approaches, most patients eventually experience relapses, frequently in association with downregulation or loss of GPRC5D expression (38–44).

**Table 2:** Emerging CAR-T and next-generation cellular therapies targeting BCMA and GPRC5D in multiple myeloma.

Brand name	Generic name	Target	Potential indication	Status
BMS-986393	Arlocabtagene autoleucel	GPRC5D	Relapsed/refractory MM, including post-BCMA CAR-T or bsAb antibody failure	Phase I/II (ongoing)
Other GPRC5D candidates (MSKCC, Janssen, others)	Talquetamab (Talvey)*	GPRC5D	Relapsed/refractory MM, including salvage after BCMA-directed therapies	Phase I/II (ongoing)
VHH CAR	Ciltacabtagene autoleucel–derived VHH constructs	BCMA	Relapsed/refractory MM	Preclinical (murine studies)
CARTyrin: CAR	CARTyrin platform construct	BCMA	Relapsed/refractory MM	Preclinical (murine studies)
D-domain CAR	Anitocabtagene autoleucel (anito-cel)	BCMA	Relapsed/refractory MM	Preclinical (murine studies)
ESO-T01	Nanobody-targeted, immune-shielded lentiviral vector (in vivo CAR delivery)	BCMA	Relapsed/refractory MM	Preclinical (mouse models); early clinical experience reported in RRMM (n = 4)

BCMA, B-cell maturation antigen; bsAb, bispecific antibody; GPRC5D, G protein-coupled receptor class C group 5 member D; MM, multiple myeloma; RRMM, relapsed/refractory multiple myeloma; VHH, variable heavy-chain domain of heavy-chain-only antibodies.*Talquetamab is a bispecific antibody, included here for contextual comparison with GPRC5D-targeted cellular approaches.

Despite these advances, the widespread efficacy of CAR T-cell therapies remains constrained by therapeutic resistance and treatment-related adverse effects. Exorbitant manufacturing costs and prolonged production timelines further restrict patient access. BCMA-targeted CAR T-cells are particularly susceptible to antigen-escape mechanisms, and the precise biological determinants underlying treatment failure in RRMM are only beginning to be elucidated (45). In parallel, serious toxicities including neurotoxicity, cytokine release syndrome, immune effector, hemophagocytic lymphohistiocytosis/macrophage activation syndrome (HLH/MAS), and secondary malignancies underscore the need for vigilant long-term and post-marketing surveillance. Notably, a distinct hypokinetic movement disorder, also referred to as movement and neurocognitive treatment-emergent adverse events, has been observed following BCMA-directed CAR T-cell therapy in MM. This syndrome was first reported in five patients enrolled in the CARTITUDE-1 trial evaluating ciltacabtagene autoleucel (cilta-cel), with symptom onset occurring weeks to months after infusion (25). Clinical manifestations included bradykinesia, rigidity, micrographia, tremor, and other Parkinsonian features. Importantly, this neurotoxicity syndrome was progressive, debilitating, and largely irreversible despite aggressive immunomodulatory and chemotherapeutic interventions (46–49). Taken together, both the remarkable clinical efficacy and the emerging limitations of CAR T-cell therapies highlight the need for complementary adoptive immunotherapy strategies. In this context, TCR-engineered T-cells expand the therapeutic landscape by enabling the targeting of intracellular antigens and neoantigens that are inaccessible to conventional surface antigen–directed approaches. Against this backdrop of antigen escape, toxicity, and limited durability associated with CAR T-cell therapies, TCR–engineered T cells have emerged as a complementary adoptive immunotherapy platform with distinct biological features relevant to MM.

## 3 Biologic Basis of TCR-engineered T-cells

### 3.1 Antigen recognition: peptide:MHC specificity

Proteasomes are essential for efficient processing and presentation of intracellular and viral proteins to generate short peptides that are presented as antigens bound to major histocompatibility complex class I (MHC-I) molecules for recognition by cytotoxic T-cells (50). TCR-engineered T-cell therapies exploit the endogenous antigen processing and presentation machinery to enable the recognition of intracellular, nuclear, and viral antigens that are inaccessible to surface restricted antigen immunotherapies. Recent early-phase clinical trials further support the feasibility of TCR-engineered T-cell therapies in MM, with evidence of antigen-specific expansion, persistence, and clinical activity (51, 52). MAGE-A1 is an antigen expressed by a subset of MM patients associated with extramedullary disease (EMD). Three different TCRs (T1367, T1405 and T1705) were isolated from three individual mice and human T-cells transduced with T1367-encoding retroviruses bind the MAGE-A1_278_-HLA-A2 multimer. MAGE-A1^+^ and HLA-A2^+^-specific cytotoxicity of T1367-transduced T cells against melanoma cells was shown. MAGE-A1-directed TCR-1367 therapy appears feasible for the tested dose in this group (52).

### 3.2 Affinity vs. avidity

Importantly, TCR-based strategies enable targeting of intracellular, transmembrane, nuclear, and viral protein–derived peptides presented by MHC-I molecules, allowing the recognition of tumor-specific and non-self-antigens with high avidity.

### 3.3 Advantages of TCR-engineered T-cells over CAR T-cells

**Figure 2:**
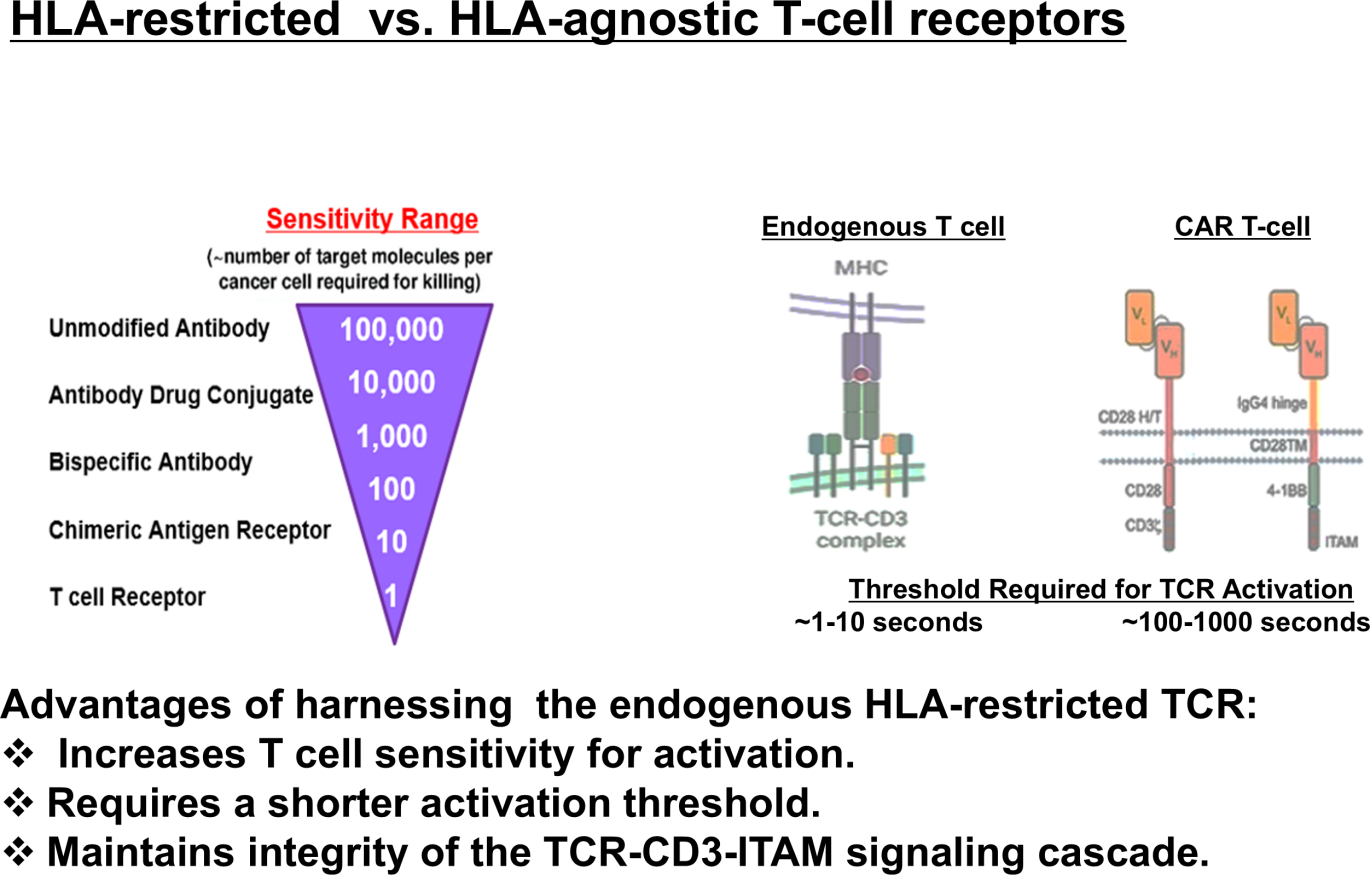
Comparison of CAR T-cell and TCR-engineered T-cell recognition and targeting mechanisms; The schematic illustrates differences between CAR T-cell therapy and TCR-engineered T cells with respect to antigen recognition, signaling, and target accessibility. CAR T cells recognize extracellular surface antigens in an MHC independent manner, whereas TCR-engineered T cells recognize intracellular, nuclear, and viral peptide antigens presented by MHC molecules.

In contrast to CAR T-cell therapy, which is limited to recognition of extracellular antigens, TCR-engineered T cells can engage a substantially broader antigenic repertoire while preserving near-physiological T-cell activation and signaling cascades that support optimal T-cell functionality. TCRs display high antigen sensitivity and can recognize tumor-associated antigens presented at low surface densities. **Figure 2** provides a schematic overview of CAR T-cell therapy, illustrating the manufacturing process and antigen-specific immune mechanisms underlying its clinical efficacy in B-cell malignancies.

T-cell recognizing human endogenous retrovirus HERV-derived antigens have been detected in patients diagnosed with hematological malignancies such as myeloproliferative disorders (MPD), chronic myelomonocytic leukemia (CMML), and acute myeloid leukemia (AML), including with low tumor mutational burden (TMB) highlighting the relevance of this target class for MM (53, 54).

Early TCR-dependent immunotherapeutic approaches, such as tumor-infiltrating lymphocytes (TILs), demonstrated clinical efficacy in selected cancers and are now approved to treat melanoma. However, in MM, TILs isolated from BM are frequently dysfunctional or exhausted due to chronic antigen exposure and immunosuppressive signals within the tumor microenvironment, limiting their therapeutic potential (55, 56). These limitations of TILs underscore the rationale for genetically-engineered TCR-based approaches that allow controlled receptor specificity and functional optimization. By enabling targeting of intracellular tumor antigens and tumor-specific NeoAgs, TCR-engineered T-cells represent a complementary adoptive immunotherapy platform that expands the therapeutic landscape beyond surface antigen–directed strategies in MM. TCR therapy may expand the use of adoptive onco-immunotherapy beyond surface antigens and enable the targeting of tumor-specific NeoAgs (46). The expanding landscape of adoptive T-cell transfer includes synthetic TCRs in addition to diverse specifications as well as currently less well studied TCR-based personalized therapy. In response, next-generation CAR and TCR-T cell therapies require programming secretion of modulatory cytokines or immune checkpoint inhibitors as well as incorporating suicide or safety switches to diminish adverse events.

### 3.4 Limitations of TCR-engineered T-cells

Despite their promise, TCR-engineered T-cell therapies share several challenges with other ACTs, including T-cell exhaustion, antigen heterogeneity, immunosuppressive tumor microenvironments, and the risk of on-target off-tumor toxicities. TCR-based approaches are also intrinsically constrained by inter-patient variability in antigen presentation, and the potential for cross-reactivity with self-peptides. Resistance mechanisms such as metabolic reprogramming, impaired antigen presentation, and tumor-intrinsic immune evasion pathways further limit durable responses. TCR-engineered T-cells are dependent on a patient’s specific human leukocytes antigen (HLA) composition that ultimately restricts therapy to HLA allele-specific populations.

## 4 Antigen processing and the immunopeptidome in multiple myeloma

### 4.1 Proteasomes and immunoproteasomes

**Figure 3:**
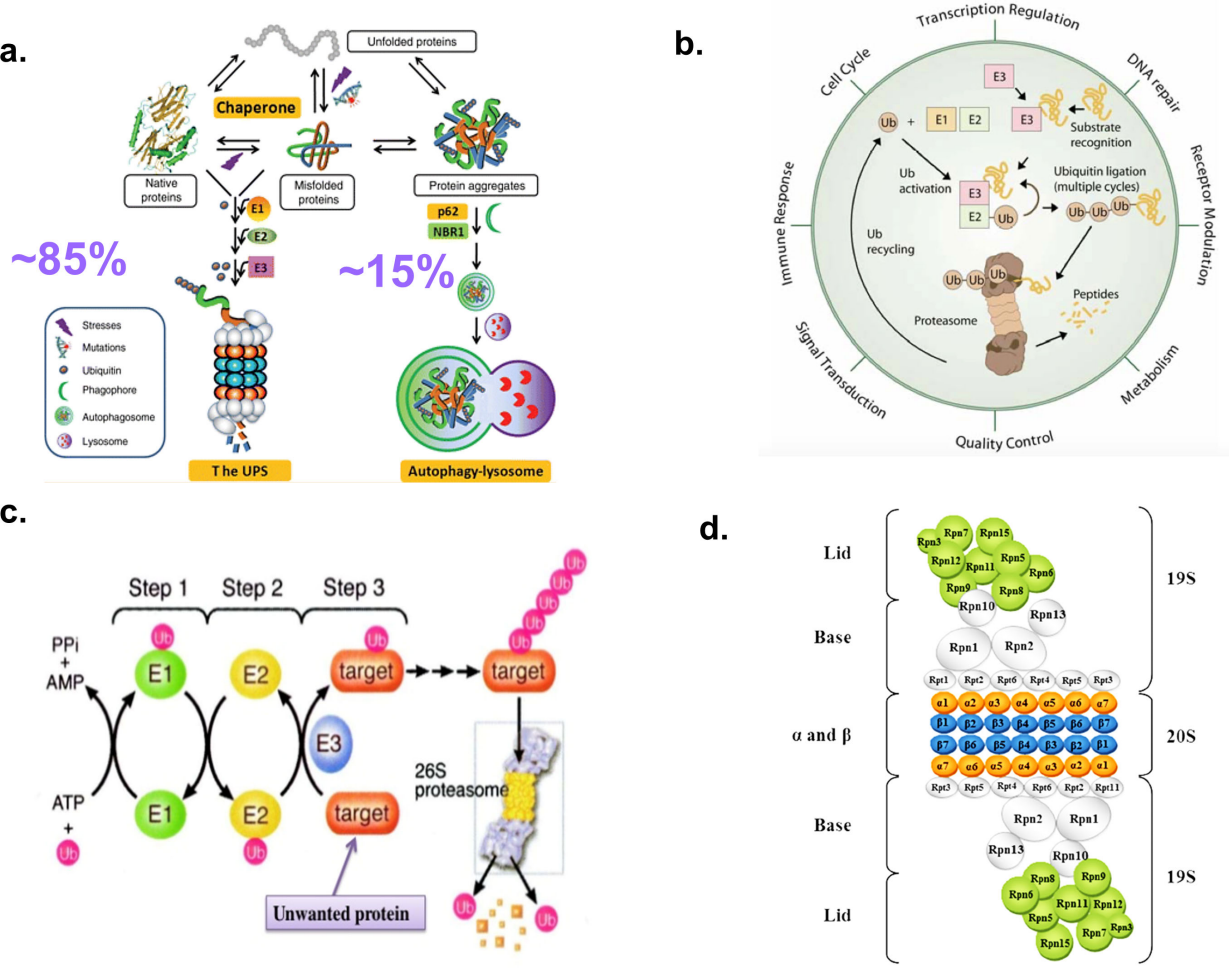
Protein degradation pathways in eukaryotic cells; **(A)** The UPS and autophagy-lysosomal pathways (57). **(B)** The UPS functions in a multitude of essential processes including regulated protein degradation, governing essential cellular pathways including cell cycle progression, apoptosis, DNA repair, and protein quality control. **(C)** Steps in UPS mediated protein degradation. Ub-mediated protein degradation is a three-step enzymatic process (tagging) followed by proteasomal destruction, ensuring selective protein removal. It involves activating, conjugating, and ligating ubiquitin to target proteins, which are then recognized and degraded by the 26S proteasome (58). **(D)** Structural view of the 26S proteasome subunits indicating the 20S catalytic core particle (CP) bound at both ends by the 19S regulatory particle (RP) (59).

Eukaryotic cells contain two primary protein clearance pathways: the Ub–proteasome system (UPS) and the lysosome–autophagy pathway (**Figure 3**). Proteasomes represent the major non-lysosomal protein degradation machinery in eukaryotic cells and regulate growth, replication, differentiation, survival, and cell death (60–63). The UPS is a highly complex, temporally controlled, and conserved pathway that regulates a multitude of essential cellular processes, including growth, replication, differentiation, survival, and cell death (**Figure 3**). The UPS consists of two coordinated processes: Ub conjugation to substrate proteins and degradation of ubiquitinated substrates by the proteasome. Ubiquitination is a three-step process: the initiating step, E1, is known as the activating step. An activating enzyme, such as UBE1 (ubiquitin-activating enzyme E1). E2 conjugation is the intermediate step; E2 is known as the conjugating step. The final step, E3, is known as the ligase step, which takes the Ub molecule from the Ub–E2 intermediate and completes the pathway by conjugating Ub to the target substrate (**Figure 3**).

**Figure 4:**
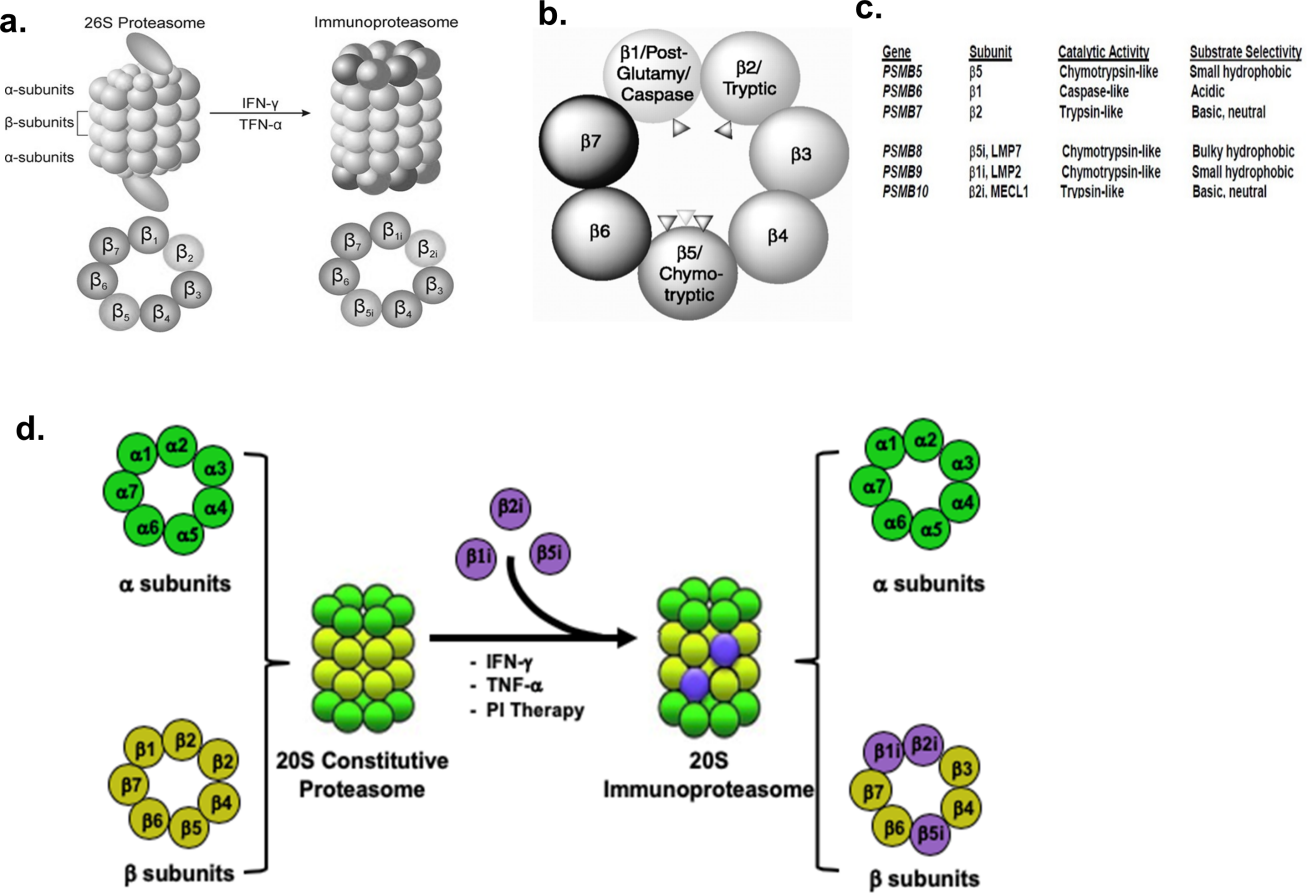
Conversion of constitutive proteasomes to immunoproteasomes; Conversion of constitutive 20S proteasomes to immunoproteasomes. **(A)** Schematic representation for conversion of the constitutive 20S proteasome to the 20S immunoproteasome. To process antigens more efficiently, proteasomes replace a subset of subunits to form immunoproteasomes (68). **(B)** Cross-sectional view of the 20S proteasome catalytic sites. **(C)** Comparison of the constitutive and immunoproteasome active sites. **(D)** Conversion of 20S constitutive proteasomes to immunoproteasomes. IFN-γ and TNF-α trigger transcriptional increases in IFN-γ that increase the expression of at least five immunoproteasome catalytic and activator subunits which cooperate to form 20S immunoproteasomes. The constitutive catalytic subunits are replaced by the immunoproteasome catalytic subunits (β1i, β2i, β5i) and activator subunits (PA28α/β).

The 26S proteasome is a ~2.4 MDa tightly regulated, multisubunit macromolecular complex that consists of a central, cylindrical 20S protein-degrading core particle (650 kDa, CP) that is capped at either or both ends by a 19S regulatory particle (RP, proteasome activator 700, PA700, 700 kDa) (**Figure 3**: 60–67). The 20S CP provides the protein-hydrolyzing activity within the 26S holoenzyme that degrades ubiquitinated proteins in an ATP-dependent process. The proteasome CPs are composed of four heptameric layers of α and β subunits stacked one above another in an α (1–7)/β(1-7)/β(1-7)/α(1-7) configuration (**Figure 4**). Within this structure, expression of processive β1i (caspase-like), β2i (trypsin-like), and β5i (chymotrypsin-like) subunits define immunoproteasomes (**Figure 4**; 69–74).

Immunoproteasomes play a central role in the degradation of intracellular proteins for the generation of antigenic peptides presented by MHC class I molecules (75, 76). They preferentially generate peptides with hydrophobic C-terminal residues, a key requirement for efficient MHC-I binding and presentation (**Figure 4**). Immunoproteasomes are constitutively expressed in antigen-presenting cells and can be induced in other cell types upon stimulation with pro-inflammatory cytokines, e.g., IFN-γ, TNF-α (**Figure 4**). Beyond antigen processing, immunoproteasomes also regulate T-lymphocyte expansion and cytokine production and are essential for immunosurveillance against malignant and virally-infected cells (75–80).

### 4.2 Peptide generation and presentation

Proteasomal generated peptide are generally 8–11 amino acids in length and contain an HLA allele-specific binding motif that defines peptide binding and assemble with MHC class I-β2-microglobulin dimers. Following proteasomal degradation, peptides are chaperoned,

loaded onto MHC-I molecules in the endoplasmic reticulum, trafficked through the Golgi apparatus, and ultimately displayed on the cell surface of all nucleated cells (81–84). Proteasome activity and antigen processing are further regulated by 11S proteasome activators PA28α, PA28β, and PA28γ (PSME1/2/3), which are evolutionarily conserved endogenous regulators of peptide hydrolysis (85–89). PA28α and PA28β form heterotetrametric complexes that associate with the 20S CP and enhance antigen processing efficiency. These regulators have evolved to optimize MHC-I antigen generation and play a central role in the antigen presentation machinery (90–96). The PA28α/β activator has been shown to increase immunoproteasome catalytic activity as well as the presentation of MHC-I antigenic peptides on MM cells. Many of these immunopeptides are in fact NeoAgs that can be potentially targeted by TCR-engineered T-cells. Hence, immunoproteasome activators represent a novel strategy to amplify and increase the myeloma immunopeptidome and broaden accessibility to T-cell therapies.

### 4.3 Risks of increased antigen presentation

While strategies aimed at enhancing antigen presentation may improve tumor immunogenicity, they also carry inherent risks. Increased presentation of self-peptides in healthy tissues may result in off-target immune activation, autoimmunity, inflammation, or tolerance induction. TCR-engineered T-cells trained to recognize tumor antigens may cross-react with structurally similar self-peptides through molecular mimicry, leading to tissue damage (97). Manipulation of antigen presentation pathways may also generate unintended neoepitopes or activate unwanted immune responses. However, recent studies have demonstrated that T-cell cross-reactivity can be mitigated without altering TCR specificity through modulation of co-signaling molecules such as CD5, CD8 and CD4 (97). A large reduction in cytotoxic T-cell cross-reactivity was observed by knocking out CD8 and expressing CD4. Cytotoxic T-cells engineered with a CD8/CD4 co-receptor switch show reduced cross-reactivity to random and positional scanning peptide libraries, as well as to self-peptides, while maintaining their on-target potency. Therefore, co-receptor switching may generate “super” selective T-cells that reduce the risk of lethal off-target cross-reactivity and offer a universal method to enhance the safety of T-cell immunotherapies for potentially any TCR. Epigenetic modifiers, e.g., HDAC inhibitors, could be used synergistically to upregulate MHC expression and antigen presentation in MM cells (93).

Proteasomes also interact transiently with multiple effectors. The ATPase valosin-containing protein (VCP)/p97 is a central component of the UPS. Together with a network of other accessory proteins, p97 functions in a wide range of intracellular processes that govern cellular physiology. The primary role of p97 may be to maintain proteasome activity under stress conditions (98, 99).

## 5 Expanding the targetable antigen repertoire

### 5.1 Strategies to enhance antigen presentation

Rationale strategies to target the immunopeptidomic landscape in tumor cells involve targeting the immunoproteasome to facilitate antigen processing and presentation, expanding and amplifying the presentation of MHC-I antigens on tumor cells (100, 101). Strategies that promote the association and binding of PA28αβ to proteasomes represent a novel strategy to increase proteasome-dependent antigen production and, therefore, will amplify and expand the repertoire of antigen presentation. Crosstalk between protein synthesis, folding, and degradation systems maintains protein homeostasis under both physiologic and pathologic conditions, e.g., exposure to oxidative stress, chemotherapy, and radiation treatment. Additional strategies to increase MHC-I antigen presentation include the use of stimulating interferons (IFNs), nanoparticle-mediated antigen delivery, and inhibiting negative regulators of MHC expression (102). Treatment of myeloma cells with IFN-γ or drugs that increase immunoproteasome catalytic activity represents a feasible, novel strategy to boost the antigen presentation machinery. Strategies that enhance antigen processing and presentation are also being developed to generate more effective cancer vaccines.

### 5.2 Neoantigens in MM (public vs. private)

Somatic mutations produce mutant proteins that are degraded to generate peptides that are complexed with MHC-I molecules and recognized as neoepitopes (103–106). However, only ~1–3% of mutation-encoded NeoAgs in individuals with solid tumors are spontaneously recognized by the endogenous T-cell pool following immunotherapy, such as TILs and immune checkpoint blockade (ICB) (107, 108). Although a large number of mutations accumulate in tumor cells, only a small fraction give rise to peptides that generate functional T-cell neoepitopes. The majority of NeoAgs (~99%) originate from patient tumor-specific mutations and are considered private (109). In contrast, public NeoAgs arise from recurrent mutations that occur in cancer drivers that are indispensable for tumor growth. NeoAgs represent a class of tumor-specific antigens generated by non-synonymous somatic mutations and are absent from healthy tissues, resulting in a low risk of autoimmunity and lack of central tolerance. NeoAg sources include point mutations, gene rearrangements, frameshifts, chromosomal translocations, chromosomal inversions, novel fusion proteins, alternative mRNA splicing, and integration of genetic material from oncogenic human endogenous retroviruses, e.g., HERVs, into the genome that are absent in non-tumor, healthy tissues.

### 5.3 Immunopeptidomics and antigen discovery

Single-antigen targeting led to immune escape and outgrowth of tumor cells *in vitro* and *in vivo*, whereas dual-antigen targeting completely cleared heterogeneous tumor-cell populations (110). Walz and colleagues described a large-scale mass spectrometry (MS) based study of the myeloma immunopeptidome identifying naturally presented HLA ligands from patient primary samples and cell lines (111, 112). Profiling comparison with benign tissues identified tumor-associated antigens that were strictly linked to the disease state. The results significantly improved antigen discovery beyond mutated neoantigen and suggest that tumor cells with a low or moderate TMB are amenable to T-cell therapies. The findings illustrate how preexisting T-cells respond to identified epitopes and highlight the value of HLA ligand-omics as a strategy to inform antigen selection and TCR-engineered T-cell design.

### 5.4 Limitations of neoantigen targeting

Although, targeting NeoAg represents an attractive strategy for TCR-engineered T-cell therapy, its application in MM is limited by the typically low-to-moderate tumor mutational burden. The scarcity of suitable immunogenic tumor-specific neo-antigens, together with the technical and logistical challenges of identifying, validating, and manufacturing TCRs against patient-specific neo-antigens, poses significant barriers to clinical translation. Consequently, NeoAg-based therapy in myeloma remains largely experimental and more feasible for patient selection or future personalized therapy supported by advances in antigen discovery and manufacturing technologies.

Recent progress in NeoAg discovery has been driven by sophisticated bioinformatic tools combined with MS-based proteomics and artificial intelligence (AI) programs. High throughput screening (HTS) and next-generation sequencing (NGS) methods have generated pipelines to rapidly and efficiently facilitate the identification of allele-specific antigenic peptides and NeoAgs (112–116). A curated list of human CD8^+^-TCR-antigens, their associated HLA ligand and TCR-directed T-cell therapies is summarized (**Table 3**).

**Table 3:** Human CD8 TCR-antigens, HLA association and TCR-directed T-cell therapies for human disease.

Peptide epitope sequence	Tumor associated antigen	HLA allele	Clinical trial number	Clinical response
SLLMWITQV SLLMWITQC	NY-ESO1 (Cancer testis antigen)	HLA-A*0201 HLA-A*02:01	NCT06889766 NCT01795976	NR
ALYVDSLFFL VLDGLDVLL	PRAME	HLA-A*02:01 HLA-A*02:01	NCT03686124 NCT03686124	NR
	MAGE			
KKLLTQHFVQENYLEY	MAGE-A3/6	HLA-DPB1*0401	NCT02111850	4 (23.5%)
NYKRCFPVI	MAGE-A4	HLA-A*24:02	UMIN000002395	0
KTWGQYWQV	GP100	HLA-A*02:01	NCT00509496	3 (19%)
YMDGTMSQV	Tyrosinase	HLA-A*02:01	NCT01586403	1 (33%)
AAGIGILTV AAGIGILTV EAAGIGILTV EAAGIGILTV	MART-1	HLA-A*02:01 HLA-A*02:01 HLA-A*02:01 HLA-A*02:01	NA NCT00509288 NCT00910650 NCT02654821	2 (13%) 6 (30%) 0 2 (16.7%)
TIHDIILECV	HPV16-E6	HLA-A*02:01	NCT02280811	
TIHDIILECV	HPV16-E6	HLA-A*02:01	NCT02280811	2 (17%)
YMLDLQPET	HPV16-E7	HLA-A*02:01	NCT02858310	6 (50%)
FLLTRILTI	HBV	HLA-A*02 or HLA-Cw0801	NCT03899415	1
FLGPLLVLQA	HBV	HLA-A*02 or HLA-Cw0801		1
KLLEIAPNC	MCPyV	HLA-A*02:01	NCT03747484	1 (25%)
NLVPMVATV	PP65	HLA-A*02:01	NCT04153279	6 (100%)
QYDPVAALF	PP65	HLA-A*24:02		6 (100%)
ATVQGQNLK	PP65	HLA-A*11:01		6 (100%)
FMNKFIYEI	AFP	HLA-A*02:01	NCT03971747	NR
HMTEVVRHC	TP53 R175H	HLA-A*02:01	NCT03412877	1 (100%)
GADGVGKSA	KRAS G12D	HLA-C*08:02	IND 27501	1 (100%)
GADGVGKSAL	KRAS G12D	HLA-C*08:02		1 (100%)
KYTKAFLV	CEBPZ	HLA-A24:02	NCT03970382	0
LLDTVLVNV	PDS5B	HLA-A02:01		0
RQFDITSVSV	SVIL	HLA-A02:01		0
DYIIRSHKV	PPP5C	HLA-A24:02		0
FVNLEQHVV	VPS13B	HLA-A02:01		0
YLHAYAKAL	GPSM2	HLA-C03:03		0
LSDAFVSV	TENM4	HLA-C05:01		0
FPNIHMETC	CLSTN1	HLA-B35:01		0
FPLDVGSIVAY	PREP	HLA-B35:01		0
LAVFPNIHM	CLSTN1	HLA-C03:03		0
RTHPGRPFF	ICK	HLA-C12:02		0
MRTHPGRPFF	ICK	HLA-B39:01		0
FVGPLDEDF	UGGT2	HLA-C12:02		0
FAFRWVLGIAY	GALNT2	HLA-B35:01		0
SISAFNLDVV	SF3A1	HLA-A02:01		0
ITADVNYNL	UAP1	HLA-A02:01		0
FEADTFFHFV	MTHFR	HLA-B40:01		0
KALDYIHHV	STRADA	HLA-C07:01		0
ALDYIHHVGYV	STRADA	HLA-A01:01		0
AGVNQISYW	CDADC1	HLA-A24:02		0
LRFLRNGLT	FCGRT	HLA-C06:02		0
EMQLTPFFI	BRPF1	HLA-A24:02		0
SLLKLGKLPL	NAALADL2	HLA-A02:01		0
ARHIHEGASL	PAPSS1	HLA-C07:02		0
FRSTRFLHI	USP34	HLA-C07:02		0
VLKNRYLVL	DDX23	HLA-B08:01		0
SRVLHKLEV	MED12L	HLA-C07:02		0
SLTQILLTWT	SDK1	HLA-A02:01		0
HMWRAAFTAER	ENOPH1	HLA-A02:01		0
LGLWYLQTALE	ROM1	HLA-A02:01		0
FQVIQPERV	RASA3	HLA-A02:01		0
IWDYVNKELA	MAN2A2	HLA-C04:01		0
VMVNHLHKY	WDFY3	HLA-A11:01		0
VSFGLQFQF	ACE	HLA-B57:01		0
KRKEPLKI	PRCC	HLA-C07:01		0
FRLIARLL	ETNK1	HLA-C07:01		0
RPSTAEKYIF	KBTBD3	HLA-B35:01		0
CYTWNQMNL	WT1 235-243	HLA-A*24:02	UMIN000011519	NED
RMFPNAPYL	WT1 126-134	HLA-A*02:01	NCT01640301	NED
IMIGVLVGV	CEA	HLA-A*02:01	NCT00923806	1 (33%)
KVAELVHFL	MAGE-A3/9/12	HLA-A*02:01	NCT01273181	5 (56%)
EVDPIGHLY	MAGE-A3	HLA-A*01	NCT01350401	0
SLLMWITQC	NY-ESO-1	HLA-A*02:01	NCT01352286	11 (44%,1 year)

Response data are shown as number of responders and percentage of evaluable patients when reported; “NA” indicates data not available, “NR ” Not reported, “0” indicates no observed responses, and “NED” denotes no evidence of disease.

## 6 Clinical experience with TCR-directed T-cells in multiple myeloma

### 6.1 Clinical trials

The clinical evaluation of TCR-engineered T-cell therapies in MM remains limited compared with CAR-T-cell-based therapies. TCR-based approaches evaluated in MM have focused on intracellular or lineage-restricted antigens presented in an MHC-I molecule, including NY-ESO-1, LAGE-1, immunoglobulin-derived peptides, and additional myeloma-associated target antigens identified through immunopeptidomic profiling. Clinical trials evaluating NY-ESO-1/LAGE-1 specific TCR engineered T-cells in patients with RRMM demonstrated the feasibility of this approach with evidence of antigen-specific T-cell persistence, and measurable clinical outcome. A subset of patients has shown durable responses particularly in context of lymphodepleting conditioning and autologous stem cell transplantation. Notably, these studies also highlighted key limitations of TCR-based therapy, including HLA restriction, antigen heterogeneity, and inconsistent durability of therapeutic responses. While BCMA-directed CAR T-cell therapies are initially effective, most patients eventually relapse, and survival after relapse remains limited. Disease recurrence has been attributed, in part, to non-uniform BCMA expressions within tumors, allowing CAR-mediated immunoediting and the outgrowth BCMA^low^ or BCMA ^(–/–)^ MM cells. Wachsman et al. developed a 2-sided model for the outgrowth of immune-escaped tumor cells after CAR T-cell or TCR-engineered T-cell treatment (110). The authors advocated dual-route targeting using HLA-dependent and HLA-independent recognition modes as a strategy to prevent immune escape. In this context, CAR T-cells can be combined with different TCRs directed to different antigens and restriction elements depending on patient characteristics and represent attractive off-the-shelf strategy.

Building on these early clinical experiences with NY-ESO-1/LAGE-1–directed TCR-based therapies in MM, a phase I/II trial evaluated the safety and activity of autologous T cells engineered to express an affinity-enhanced T-cell receptor (TCR) recognizing a naturally processed peptide shared by the cancer-testis antigens NY-ESO-1 and LAGE-1 (117). Twenty patients with antigen-positive MM received an average 2.4 × 10^9^ engineered T-cells 2 days after ASCT. Infusions were well tolerated without clinically apparent cytokine-release syndrome, despite high IL-6 levels. Engineered T-cells expanded, persisted, trafficked to BM and exhibited a cytotoxic phenotype. Persistence of engineered T-cells in blood was inversely associated with NY-ESO-1 levels in BM. Disease progression was associated with loss of T-cell persistence or antigen escape, in accordance with the expected mechanism of action of the transferred T cells. Encouraging clinical responses were observed in 16 of 20 patients (80%) with advanced disease, with a median progression-free survival of 19.1 months.

Recent efforts have focused on broadening the repertoire of targetable myeloma antigens using mass spectrometry-based HLA ligandome profiling and advanced next-generation TCR discovery platforms. Several TCR directed therapies targeting shared or public antigens are ongoing in early clinical development or active clinical trials for MM, reflecting renewed interest in this modality as a complement or alternative to CAR-based therapies. Available clinical data supports the biological feasibility of TCR-engineered T-cells in MM while emphasizing the need for optimized antigen selection, improved T-cell persistence, and strategies to counteract immune evasion in the BM microenvironment.

In addition to selecting appropriate target antigens and generating therapeutic TCRs, the preclinical analysis of TCRs is a critical bottleneck because it is essential to ensure safe and efficient application of TCR-T-cells (118). A recent pipeline screened TCR-T-cells for recognition of unintended HLA alleles, and a syngeneic, HLA-A*02:01-transgenic mouse model used as a proxy for off-target reactivity *in vivo*. The identification of candidate therapeutic TCRs would benefit from a widely applicable strategy. Strategies to employ antigen-specific, *ex vivo* expanded T-cells that traffic to and persist in BM, are well tolerated, and produce promising response rates when infused after stem cell transplantation (119).

### 6.2 Safety considerations

Increasing antigen presentation can lead to off-target effects like autoimmunity (T-cells attacking healthy tissues due to cross-reactivity with similar self-antigens), inflammation, or tolerance induction.,. This strategy carries a high risk of off-target toxicity if they lead to the presentation of self-peptides in healthy tissues. T-cells trained to recognize tumor antigens might cross-react with similar peptides in normal tissues, trigger autoimmunity and cause tissue damage. Modifying antigen presentation, e.g., with aggregated antibodies, can increase uptake by dendritic cells (DCs), potentially generating neoepitopes and activating T-cells against unintended targets. TCR-engineered T-cells might recognize unintended self-antigens with similar TCRs due to structural similarities through molecular mimicry.

## 7 Engineering challenges and optimization strategies

### 7.1 TCR chain mispairing

Following genetic engineering, the recombinant TCR α chains (TRAC) and β chains may bind with existing endogenous chains, giving rise to unintended or self-reactive receptors. One mitigation strategy is TRAC knockout (KO) using CRISPR/Cas9 to eliminate native TCRs, preventing mispairing, reducing alloreactivity or GVHD, and improving TCR expression and function. TCR KO and replacement strategies can generate more potent and less variable T-cells and are particularly relevant for allogeneic applications (119). Another area that remains insufficiently explored is antigen spreading following TCR-engineered T-cell therapy. It remains unclear whether TCR-T–driven tumor cell killing can induce secondary immune responses against additional tumor-associated antigens beyond the initially targeted epitope. Understanding antigen spreading may be critical for enhancing response durability and limiting antigen escape.

T-cells are valuable tools in onco-immunotherapy based upon their ability to bind tumor-specific antigens and the capacity to specifically eliminate cancer cells. T-cells genetically modified *ex vivo* to express cancer-specific NeoAg-specific TCR, unlike CARs, recognize MHC-I-presented peptides derived from human or viral polypeptides that have arisen from diverse cellular compartments. However, TCR-engineered T-cells remain less clinically advanced than other T-cell–directed therapies, including bispecific T-cell engagers and next-generation armored CAR T-cells.

### 7.2 T-cell metabolic constraints

Cellular metabolism plays a critical role in regulating T-cell function within the tumor microenvironment (TME). Lactate, a key glycolytic metabolite, has been shown to increase the stemness of CD8+ T cells and augment anti-tumor immunity (120, 121). To fulfill the bioenergetic demands of activation, T-cells rely on aerobic glycolysis, converting glucose to lactate rather than oxidative mitochondrial metabolism. The functional activity of TCR-engineered T-cells may be finely tuned through modulation of glycolytic activity. TCR activation also induces rapid glutamine uptake, and glutamine deprivation within the TME may limit the efficacy of both T-cell–based and TCR-engineered therapeutic approaches (122, 123).

### 7.3 Tumor microenvironments effects on TCR-engineered T-cells

The efficacy of TCR-engineered T cells depends on sustained functional activity, yet the mechanisms underlying T-cell exhaustion and dysfunction remain poorly defined. The BM microenvironment drives T-cells toward terminal differentiation, exhaustion, and dysfunction through chronic antigen exposure and sustains immunosuppressive cues (124). Myeloma-associated T-cells frequently express inhibitory receptors such as PD-1, TIM-3, and LAG-3, with impaired proliferative and effector capacity. The activity of TCR-engineered T-cells may also be limited by variable peptide–MHC presentation on myeloma cells due to differences in antigen processing, HLA expression levels, and tumor heterogeneity (56, 125).

### 7.4 T-cell fitness

In elderly MM patients, age-associated T-cell fitness is diminished, resulting from extensive prior therapies and heterogeneous variability in antigen presentation. These factors should be incorporated into the therapeutic framework for TCR-engineered T-cell therapy. In this context, T-cell exhaustion arises from chronic antigen exposure and sustains immunosuppressive cues within the BM microenvironment. Myeloma-associated T cells frequently display a distinct exhausted phenotype characterized by increased expression of inhibitory receptors, including PD-1, TIM-3, and LAG-3, along with impaired proliferative and effector capacity. These exhausted states pose a critical barrier to achieving durable TCR-engineered T-cell responses and highlight the need for strategic approaches aimed at limiting or reversing exhaustion, including optimized receptor design, combinatorial checkpoint blockade, or modulation of the BM niche (126, 127).

NeoAgs represent an attractive approach for TCR-engineered T-cell therapy, however, their application in MM is limited by the typically low-to-moderate tumor mutational burden (TMB). The paucity of suitable immunogenic tumor-specific NeoAgs, together with technical and logistical challenges associated with identifying, validating, and manufacturing TCRs directed against patient-specific private NeoAgs, poses significant barriers to widespread clinical translation. Consequently, NeoAg-based therapy remains experimental in MM, although MS-based immunopeptidomics combined with AI-driven approaches may enable identification of both public and private NeoAgs for future personalized therapies. Despite current logistical and financial barriers, highly personalized TCR-T therapies may ultimately prove most beneficial when applied earlier in the disease course, particularly as understanding of relapse mechanisms, patient selection, and immune fitness continues to improve.

## 8 Future directions in TCR-based therapy

### 8.1 Bispecific T-cell engagers

Bispecific T-cell engagers offer off-the-shelf availability and have demonstrated meaningful clinical outcomes; however, their efficacy is highly dependent on the exhaustion state of the T-cells. Bispecific T-cell engagers also remain restricted to targeting surface antigens, making them susceptible to antigen escape. Armored CAR-T cells may enhance T-cell persistence and overcome resistance to immunosuppression, but they also maintain certain CAR-specific limitations, including surface antigen dependence and increased immune-mediated toxicity risk. In contrast, TCR-engineered T-cells enable targeting of intracellular, extracellular, nuclear, and viral antigens, thereby expanding the repertoire of therapeutically actionable targets beyond what is accessible to bispecific T-cell engagers and CAR T-cell therapies (128). Lineage-restricted and tumor-specific antigens presented by the MHC-I system further expand this therapeutic space. Although clinical outcomes in MM remain constrained by HLA restriction and antigen presentation variability, advances in TCR discovery platforms support their development as a complementary strategy, particularly in patients who relapse after surface antigen–directed therapies.

### 8.2 Gamma-delta T-cells

Gamma-delta (γδ) T-cells show strong potential as a therapeutic modality for MM based on their ability to directly recognize and kill cancer cells independent of MHC presentation, making them promising off-the-shelf cellular therapies. The γδ T-cells constitute ~5%–10% of peripheral blood lymphocytes, perform a key role in tumor immunosurveillance, and mediate anticancer responses through direct and indirect mechanisms. The primary obstacle to the development of γδ T-cell immunotherapy has been the difficulty in expanding this relatively rare T-cell subset to clinically relevant numbers. Although exhaustion in conventional αβ T-cells is well characterized, emerging evidence suggests that canonical exhaustion markers such as PD-1 may not define exhaustion in γδ T-cells as functional Vδ1 cells often express high levels of PD-1. Importantly, clinical trials involving allogeneic γδ T-cells have not reported graft-versus-host disease (GVHD), supporting the feasibility of off-the-shelf approaches (129).

### 8.3 TCR-engineered T-cells as early forms of therapy

The challenge of HLA restriction is often underestimated, as each TCR construct is compatible only with patients expressing the corresponding HLA allele. HLA diversity increases clinical screening burden and manufacturing complexity, limiting widespread application of TCR-engineered T-cell therapies. Since TCR-engineered T-cells may require genetic modification of autologous T-cells, it may be beneficial to initiate therapy as early as possible before autologous T-cells have been eliminated or exhausted by prior treatments. Chemotherapy significantly impacts T-cells, often causing depletion, dysfunction, exhaustion, metabolic damage, and altered subset composition, thereby weakening immune competence. CAR T-cells also become exhausted or receive inhibitory signals that limit their activity.

## 9 Conclusions

The past two decades have witnessed rapid advancement in understanding the basic mechanisms that underly antigen presentation and the antitumor function of various types of immune cells. These findings have led to the development of adoptive cellular therapies as platforms for cancer immunotherapeutics. TCR-directed T-cells offer the capacity to target an expanded range of tumor neoantigens by recognizing HLA-presented peptides, unlike CAR T-cells which are limited to only surface antigens. TCR-directed T-cells also provide high specificity, enhanced tumor infiltration in solid tumors, superior sensitivity, and the potential for long-term, durable immune responses. Bottlenecks include the development and implementation of cutting-edge immunopeptidomic and sequencing approaches combined with bioinformatic pipelines to identify and prioritize public and private neoantigens. Future directions include the development of high-throughput methods to identify tumor-reactive TCRs for the expeditious, scalable expansion of autologous or allogeneic lymphocytes.
